# Revolutionizing Pediatric Neurophysiology With Magnetoencephalography

**DOI:** 10.1111/psyp.70262

**Published:** 2026-04-12

**Authors:** J. C. Edgar, T. W. Wilson, S. Taulu, A. C. Nugent, S. P. Ahlfors, T. Kenet, Y. Chen, A. N. Bosseler, H. L. Green, E. Heinrichs‐Graham, W. Gaetz, E. Larson, F. T. Candelaria‐Cook, N. M. Bell, E. M. Davenport, B. Howell, M. Evans, B. Ahtam, M. X. Huang, T. W. Ward, B. Share, M. Mody, J. J. Son, D. Cellier, N. A. Peatfield, T. Bardouille, L. M. Bailey, S. Grewal, K. Pratt, S. Knappe, O. Alem, G. A. Miller, J. Stephen

**Affiliations:** ^1^ Lurie Family Foundations MEG Imaging Center, Department of Radiology The Children's Hospital of Philadelphia Philadelphia Pennsylvania USA; ^2^ Department of Radiology, Perelman School of Medicine University of Pennsylvania Philadelphia Pennsylvania USA; ^3^ Institute for Human Neuroscience Boys Town National Research Hospital Boys Town Nebraska USA; ^4^ Department of Pharmacology and Neuroscience Creighton University Omaha Nebraska USA; ^5^ Institute for Learning and Brain Sciences University of Washington Seattle Washington USA; ^6^ Department of Physics University of Washington Seattle Washington USA; ^7^ MEG Core Facility, National Institute of Mental Health National Institutes of Health Bethesda Maryland USA; ^8^ Athinoula A. Martinos Center for Biomedical Imaging, Department of Radiology Massachusetts General Hospital and Harvard Medical School Boston Massachusetts USA; ^9^ Department of Neurology Massachusetts General Hospital, Harvard Medical School Boston Massachusetts USA; ^10^ The Mind Research Network a Division of Lovelace Biomedical Research Institute Albuquerque New Mexico USA; ^11^ Department of Radiology University of Texas Southwestern Medical Center Dallas Texas USA; ^12^ Fralin Biomedical Research Institute at Virginia Tech Carilion Roanoke Virginia USA; ^13^ Department of Human Development and Family Science Virginia Tech Blacksburg Virginia USA; ^14^ Translational Biology, Medicine, and Health Program Virginia Tech Roanoke Virginia USA; ^15^ Department of Pediatrics Boston Children's Hospital, Harvard Medical School Boston Massachusetts USA; ^16^ Department of Radiology University of California San Diego California USA; ^17^ ANT North American, Inc. Nashville Tennessee USA; ^18^ Department of Cognitive Science University of California, San Diego La Jolla California USA; ^19^ HeyBro, Inc. Vancouver British Columbia Canada; ^20^ Department of Physics & Atmospheric Science Dalhousie University Halifax Nova Scotia Canada; ^21^ Department of Neurology The Children's Hospital of Philadelphia Philadelphia Pennsylvania USA; ^22^ Tristan Technologies, Inc. San Diego California USA; ^23^ Paul M. Rady Department of Mechanical Engineering University of Colorado Boulder Boulder Colorado USA; ^24^ FieldLine Industries Boulder Colorado USA; ^25^ FieldLine Medical Boulder Colorado USA; ^26^ Department of Psychology University of California Los Angeles California USA; ^27^ Department of Psychiatry and Biobehavioral Sciences University of California Los Angeles California USA; ^28^ Department of Psychology and Beckman Institute University of Illinois Urbana‐Champaign Urbana‐Champaign Illinois USA

## Abstract

This paper considers the current and future use of magnetoencephalography (MEG) for assessing neural activity in children (birth to 18 years old), including the well‐established use of SQUID (Superconducting QUantum Interference Device) MEG technology as well as the very rapidly developing Optically Pumped Magnetometry (OPM) technology. A primary conclusion is that the changing landscape of pediatric neurophysiology studies foretells a revolution in electromagnetic neuroimaging. These changes will produce some discontinuity, progressing away from what once worked well enough, namely, examining neural activity at the level of the EEG or MEG sensor, but is not up to current and anticipated challenges. Given features intrinsic to MEG, including simpler mathematical models for source localization and higher‐dimensional representation of neural activity, little effect of open fontanelles and sutures on infant neural measures, and reference‐free neural measures, MEG will often be the preferred method for assessing neural activity in children. In particular, noninvasive, whole‐brain MEG sensor data with source localization provide measures of neural activity in brain space that richly represent the structure and maturation of neural activity spanning both local and regional processes, as well as measures of connectivity within and between brain regions. Assessing neurophysiology in brain space is also essential for associating local neural activity with local brain structure (e.g., gray and white matter) and brain chemistry (e.g., magnetic resonance spectroscopy data). It is also highly likely that MEG data are more future‐proof than EEG data (higher dimensionality, ease of source localization), especially for advanced source localization methods as well as advanced analysis methods yet to be developed and applied to previously collected data. The emergence of OPM‐based MEG opens a new age of imaging for children and infants, such as translating the source localizing abilities of MEG in adults to wearable systems in young children. Looking forward, greater access to MEG and other advanced imaging technologies, the accessibility of greater computational power, and the rapid development of open‐source software will combine to improve our methods and inform our research questions, all leading to a better understanding of how the human brain changes and supports behavioral development from birth to adulthood.

## Impact Statement

This paper considers the current and future use of magnetoencephalography (MEG) for assessing neural activity in children (birth to 18 years old), including the well‐established use of SQUID (Superconducting QUantum Interference Device) MEG technology, as well as the very rapidly developing Optically Pumped Magnetometry (OPM) technology. A primary conclusion is that the changing landscape of pediatric neurophysiology studies foretells a revolution in electromagnetic neuroimaging. These changes will produce some discontinuity, progressing away from what once worked well enough, namely examining neural activity at the level of the EEG or MEG sensor, but is not up to current and anticipated challenges.

## 1 Introduction

In the spring of 2023, 2024, and 2025, several pediatric brain imaging scientists from across North America (the coauthors on this paper) met to consider the current and future use of magnetoencephalography (MEG) for pediatric research in those under 18 years old. This paper reports on the key themes discussed at these 3‐day retreats, including the well‐established use of SQUID (Superconducting QUantum Interference Device) MEG technology and the rapidly developing optically pumped magnetometry (OPM) approaches. In this paper, we demonstrate how advanced electromagnetic brain imaging provides an understanding of neural activity in children that is more detailed than previously possible. As described throughout this paper, given its inherent advantages, MEG will often be the preferred method for studying neural activity in children.

One primary goal of pediatric brain imaging studies should be to increase the precision of our measure of neural activity. Analyses of EEG and MEG sensor data that are limited to scalp‐level measures are increasingly not scientifically satisfactory, as the provided results are difficult or impossible to interpret in a way that would expand our understanding of neural phenomena or open up new territory. For example, when describing the maturation of resting‐state neural activity, acceptable solutions should not look only for scalp‐region differences in sensor space. Rather, and as Sections 2.2 and 2.3 describe in detail, acceptable solutions will demonstrate differences in maturation via reporting on neural activity in brain space (i.e., describing maturation within and between brain areas). Similarly, acceptable solutions will usually not involve reporting on how EEG or MEG sensor activity, which almost always contains superimposed contributions from neural activity in multiple brain regions, changes across child development. The measures that best assess brain area‐specific neural activity should be those that inform our understanding of neural activity in children. In most cases, these will be measures of brain activity in source space. Although for some purposes sensor‐level analysis will continue to contribute in clinical and research contexts, making brain source analysis the standard in clinical and research contexts will produce some disruption and discontinuity, yet the case for it is compelling, as the rewards will certainly exceed the costs.

This report is divided into five parts. Section 2.1 makes the case for examining neural activity in pediatric populations. Section 2.2 describes the benefits of MEG relative to EEG for assessing neural activity in children. Section 2.3 considers resting‐state neural activity to provide an example of the need for data with a high information content (here referencing work demonstrating higher dimensional data for MEG than EEG) and good spatial resolution of source localization (here detailing problems associated with examining sensor measures) to improve our understanding of neural activity in children in a way that would substantially advance the field. Section 2.4 considers the cost of neurophysiology research, in terms of both money spent and amount of information acquired, the perceived difficulty of conducting multisite MEG studies, and the need for more MEG sites as well as more scientists trained in the use of MEG and EEG source localization methods. Section 2.5 considers the future of OPM MEG as a potentially revolutionary improvement in research and clinical practice.

## 2 Electrophysiology in Pediatric Populations

### 2.1 Examining Neural Activity in Children

The shift in research strategy in the developmental neurophysiology literature over the last two decades, from primarily studying adult populations to more often studying pediatric populations, is occurring both at the level of individual laboratories and at the level of nations, with large pediatric brain imaging programs sponsored by governments such as the ChildBrain project in the European Union (https://cordis.europa.eu/project/id/641652), the Adolescent Brain Cognitive Development study in the United States (ABCD; https://abcdstudy.org/), the Province of Ontario Neurodevelopmental Disorders Network in Canada (POND; https://pond‐network.ca), and the more recent HEALthy Brain and Child Development Study in the United States (HBCD; https://heal.nih.gov/research/infants‐and‐children/healthy‐brain). The rationale for these endeavors is articulated in the NIMH 2023 Strategic Plan (NIMH Strategic Plan for Research 2023), in which Goal 2 notes:

Most mental illnesses first present in childhood or adolescence, yet mental illnesses are likely the late behavioral manifestations of changes that began years earlier. These early alterations may influence the course of brain and behavioral development and establish the trajectories of mental illnesses. To better understand these trajectories, we need to develop a comprehensive picture of typical and atypical brain and behavioral development across the lifespan.

The urgency to understand typical and atypical early brain development is further underlined by the emergence of the Developmental Origins of Health and Disease (DOHAD; https://www.ncbi.nlm.nih.gov/pmc/articles/PMC5856182/) field, which has established that early environmental insults increase the risk of adult neurodegenerative diseases such as Alzheimer's and Parkinson's disease and has also found that these insults may originate in utero.

Although we have learned an impressive amount about the brain from examining hemodynamic signals with fMRI (Bernas et al. 2018; Easson and McIntosh 2019; Delbruck et al. 2019; Kardan et al. 2022; Zhang et al. 2019), there is increasing agreement among neuroscientists that understanding brain function requires deep insight into the millisecond‐level dynamics of neural function. In large part, this is because the brain processes information via electro‐chemical signaling that typically occurs on the order of milliseconds. In line with NIMH's Research Domain Criteria (RDoC) initiative (Cuthbert et al. 2023), the NIMH 2023 Strategic Plan prioritizes studies examining the circuits involved in complex behavior using high spatial and temporal resolution techniques (Goal 1). The urgent need for studies that noninvasively assess neurophysiological function in typically developing as well as patient populations is also noted. For example, NIMH 2023 Strategic Goal 1 prioritizes, “Developing novel, age‐appropriate imaging assays with higher spatial and temporal resolution for visualization and analyses of brain structure, maturation, connectivity, and function, with particular emphasis on advancing real‐time measurement approaches” (Strategy 1.1.C.3) and “Conducting brain‐wide analyses to determine which neural circuits drive network patterns associated with a pathology” (Strategy 1.3.A.1). Given such goals, the assessment of anatomically localized activity has significant advantages relative to raw sensor measurements, which, as discussed below, problematically include activity from many different brain areas (even a spatially focal source in the brain produces a wide‐spread pattern of scalp potentials and magnetic fields; this is often referred to as “volume conduction” in EEG) (Nunez and Srinivasan 2006; Srinivasan et al. 2007; Baillet et al. 2001). Among other advantages, source activity mapping facilitates the discovery of an individual's neural fingerprint (e.g., resting‐state neural connectivity patterns that distinguish individuals within a cohort (Da Silva Castanheira et al. 2021), measures that are of interest for personalized diagnosis and treatment) as well as control and case group differences that are region‐specific (Da Silva Castanheira et al. 2024; Edgar, Heiken, et al. 2015).

Invasive electrophysiology, utilizing electrodes placed directly in the brain or on the brain surface, provides remarkable richness of data. However, such data can only be obtained from patients undergoing surgery. The present paper only addresses noninvasive electromagnetic methods (EEG and MEG) in pediatric populations (NIMH Strategy 1.3.A.1). We focus on the use of MEG to measure brain activity, based on evidence that MEG methods have advanced to where they can uniquely contribute to our understanding of neural activity (Sadaghiani et al. 2022). Measures of interest include neural activity in the time domain (e.g., evoked components) and the time–frequency domain (e.g., intertrial coherence, cross‐frequency coupling, and total power measures). Source‐space analyses in both domains offer far richer information than traditional sensor‐space analyses, such as measures of functional connectivity that can be examined both locally (e.g., alpha‐to‐gamma phase‐amplitude coupling within a specific brain area or inter‐region connectivity) and globally (e.g., frequency‐based connectivity and graph‐theory analyses) (Baillet 2017; Wilson et al. 2016; Brookes et al. 2016, 2011; Hunt et al. 2019). As methods for assessing neural activity continue to evolve, offering increasingly detailed insights into neural processes, MEG data are particularly well‐suited for these advanced analyses.

Multimodal studies are also increasingly used to assess how brain structure and brain chemistry are related to neural function. For example, pediatric studies increasingly report associations between brain structure (e.g., structural MRI) or chemistry (e.g., MRS) and brain function (e.g., EEG or MEG) (Edgar, Heiken, et al. 2015; Green et al. 2022; Berman et al. 2023; Stephen et al. 2021; Papadelis et al. 2014; Hunold et al. 2014; Urbain et al. 2019), including longitudinal studies examining the co‐maturation of brain structure and function (Candelaria‐Cook et al. 2022; Chen et al. 2024). Such multimodal studies are crucial to understanding normative brain development (e.g., how maturational changes in white matter and neural processing speed are related) and will surely help identify brain abnormalities and inform potential treatments in patients. Multimodal assessment of brain structure and function in longitudinal pediatric samples also informs adult studies, as the longitudinal study of neurodevelopment provides a unique opportunity to determine causality in the linkages between structure and function as cognitive abilities emerge across childhood. Longitudinal (and cross‐sectional) studies also provide the ability to identify age‐specific neurophysiology measures, with some of the EEG and MEG phenomena investigated in adults not present in infants and young children (Edgar, Blaskey, et al. 2020; Edgar et al. 2016; Bosseler et al. 2021, 2024, 2013; Kuhl et al. 2014; Zhao et al. 2021). In addition, studies investigating neurodevelopmental disorders have shown divergent developmental trajectories in patients versus typically developing populations (e.g., figures 2 and 3 in Mamashli et al. 2018, figure 4 in Alho et al. 2021, figure 3 in Safar et al. 2021, and for a review Rhodes et al. 2024). Multimodal studies will greatly benefit from source‐space analyses as methods such as MRI and MRS yield results that are naturally in source space, meaning that a faithful correlation of results across methodologies can only take place if the MEG/EEG results are also mapped into source space.

In sum, there is broad consensus in the field that if we want to understand brain activity in children, then we must understand how populations of neurons function with millisecond temporal precision (i.e., the neural timescale), locally within specific brain areas, as well as across different brain areas. As detailed below, progress in this area is best accomplished via studying brain activity in anatomically localized source space rather than in sensor‐space signals.

### 2.2 Noninvasively Assessing Neural Activity in Children Using EEG and MEG ^1^

**FIGURE 1:**
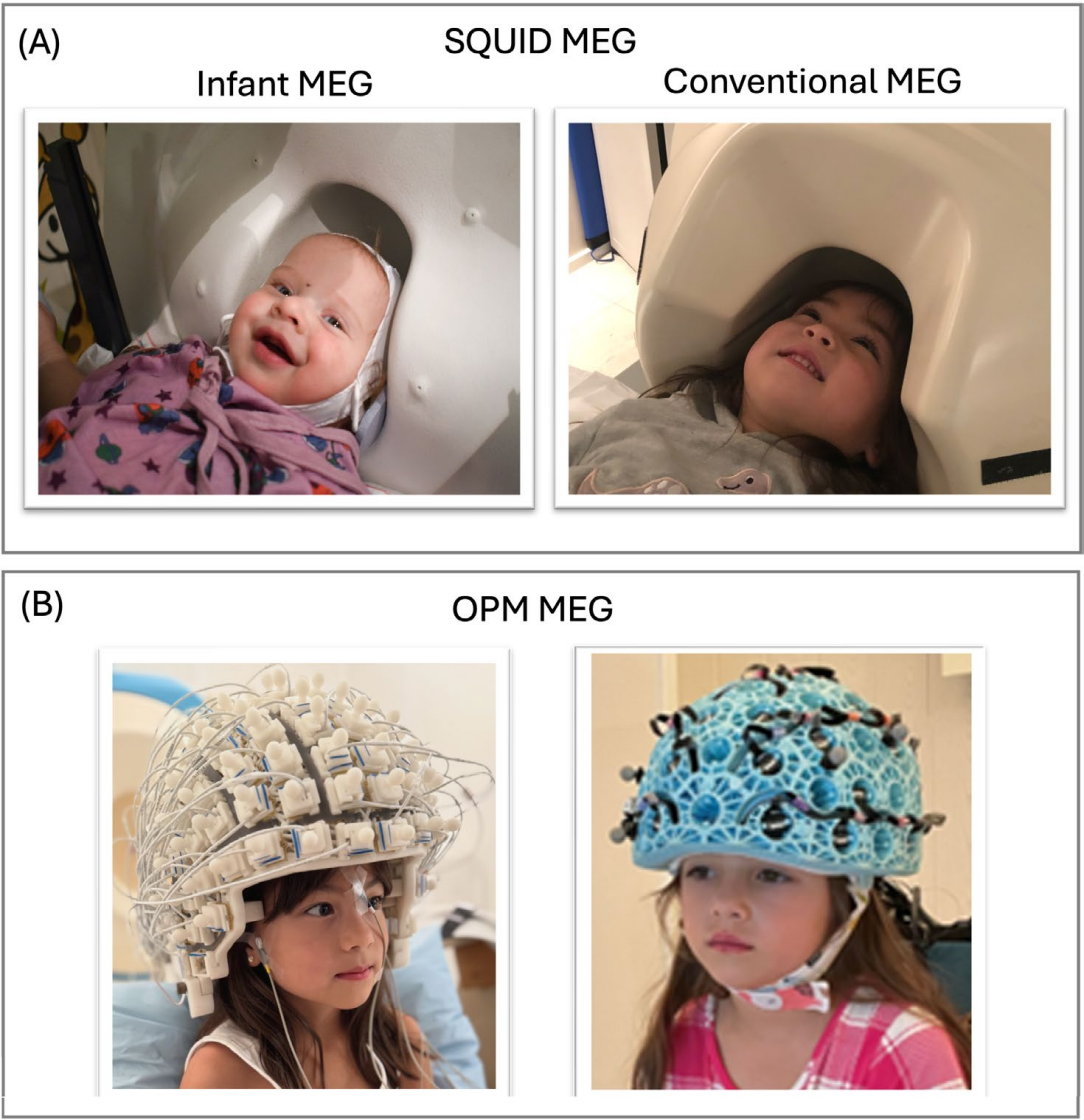
Photographs of MEG systems: (A) Left: SQUID MEG Artemis 123‐channel system (Tristan Technologies Inc.); Right SQUID MEG 275‐channel system (CTF MEG Neuro Innovations Inc.), (B) Left: OPM MEG 128‐channel (FieldLine Medical Inc.); Right: OPM MEG 80‐channel system (QuSpin, Colorado, USA) mounted in a rigid wearable helmet (Cerca Magnetics Ltd. Nottingham, UK), Cerca image from Rhodes, Rier, Singh, et al. (2025) and used with permission.

Figure 1 shows examples of SQUID and OPM MEG systems. MEG and EEG are safe, quiet, and straightforward methods to noninvasively study neural function with high temporal resolution. This section considers the relative advantages of MEG and EEG with respect to measuring neurophysiological activity in pediatric populations. MEG and EEG instrumentation and analysis methods are not discussed in this paper (seminal papers and books on this topic are provided in Endnote 1).

In the context of the goal of evaluating brain activity in anatomically localized source space, below we make a case for the use of MEG in pediatric studies, emphasizing advantages of MEG related to the physics and mathematics of signal generation (i.e., the so‐called “forward problem” of determining how neural activity produces externally measured scalp potentials (EEG) and magnetic fields (MEG)). Although both EEG and MEG signals have their origin mainly in the dendritic currents in pyramidal neurons, the magnetic field detected with MEG has several properties that simplify the interpretation of the data in terms of neural source activity in the brain. The important practical question of the availability and cost of EEG and MEG is addressed in Section 2.4.

A premise for the case for MEG is that neural function characterized at the most fundamental level available is an essential component of mapping systems‐level phenomena. To this end, MEG scientists often seek to obtain measures of neural activity in “brain space”, using source‐localization methods to transform the extracranial signals to estimated spatiotemporal patterns of activity within the brain. One important reason to focus on brain space is that sensor measures typically contain brain activity from many different brain areas (the superposition of brain activity (Orrison 1995; Edgar et al. 2003), see examples in Section 2.3), as well as nonbrain noise such as electromagnetic activity from face and neck muscles, eye‐blinks, and heartbeat, and these signals are often difficult to separate. As such, analysis methods that are less susceptible to this problem are much preferable.

In infants, EEG and MEG exams are relatively well tolerated (especially compared to MRI exams) and can be performed when infants are awake or asleep. The recent availability of high‐density infant EEG sensor nets has greatly improved the topographic resolution of EEG and the feasibility of such recordings. However, EEG is strongly dependent on the different conductivities of various tissues (e.g., brain, cerebral spinal fluid, scalp, and skull) (Flemming et al. 2005; Lew et al. 2013; Lau et al. 2011). Furthermore, distortions of scalp potentials due to open fontanels and sutures in younger infants further add to the challenges inherent in EEG.

The conductivity values needed for EEG source localization are not only difficult to determine in infants but change during infant development due to skull maturation (Flemming et al. 2005; Lew et al. 2013). In contrast, as detailed in Okada et al. (Okada et al. 2016), the magnetic field outside of the scalp produced by neuronal currents is only weakly affected by inhomogeneities and anisotropy of the electrical conductivity within the head (Geselowitz 1970; Geselowitz and Miller 1973; Plonsey 1981), a fundamental advantage for the use of MEG in participants of all ages. The magnetic field is also relatively unaffected by the fontanels and sutures that provide paths of high electrical conductivity through unfused cranial bones (Lew et al. 2013, 2021; Lau et al. 2011; Barth et al. 1986; Okada et al. 1999). Crucially for infant clinical and research studies, and especially those with longitudinal designs, MEG is thus largely unaffected by skull maturation. These properties provide notable advantages for MEG over EEG with respect to the accuracy of the forward model and thus for source localization in general (Hämäläinen et al. 1993), thus providing more valid mapping of such neural activity at any age, especially as the brain and skull mature early in life.

In addition to this pervasive structural biophysical advantage, a second advantage of MEG over EEG is the nature of the raw sensor data. In EEG, scalp potential recordings are always differential, requiring a choice of a reference electrode and thus an indirect measure of the electrical field. In contrast, in MEG the magnetic field can be recorded directly at a single location, without requiring a reference measurement. The reference issue complicates the practical interpretation of EEG sensor waveforms. However, the choice of the reference electrode does not affect source localization analyses (Michel and He 2019), further emphasizing the benefits of analyzing data in brain space (for discussion of issues associated with the choice of EEG reference and inference about brain function, see an editorial by Bringas Vega et al. (2019) and the associated collection of papers).

A third advantage of MEG over EEG is the richness of MEG data, demonstrated in a recent paper by Taulu and Larson (2021). Comparing the properties of electrical and magnetic recordings, Taulu and Larson showed that there is inherently less spatial information available in EEG than in MEG data. They noted (999): “…even without considering any effects caused by conductivity boundaries [and the much greater distortions they create in EEG than MEG], the little spatial information in the measured EEG signal can be understood by the fact that the electric potential is always a proportional quantity between two points.” This means that it is difficult to recover the finer spatial details of the underlying source currents. Furthermore, Petrov and Sridhar (2013) noted that the information content of the resulting spatially sampled EEG scalar field tends to be less than when sampling the underlying vector field (the electric field), even though in principle the electric field can be derived from the electric potential in the continuous case. This observation is in line with their simulation study indicating the potentially better resolution achievable by a hypothetical electric field measurement. In contrast, MEG records magnetic fields, comparable to electric fields rather than to electric potentials, thus providing an inherently superior representation of the neural phenomena.

Judging the dimensionality (information content) of MEG and EEG data for whole‐head scalp recordings, Taulu and Larson (2021) estimated up to 80 degrees of freedom for high‐density MEG versus less than 40 degrees of freedom for high‐density EEG. They then examined actual auditory evoked response data recorded with a 60‐channel EEG system. They showed that, using just the 21 sites of the standard 10–20 EEG electrode montage, they could explain more than 93% of the 60‐channel auditory response variance. Thus, the 21‐channel montage yields essentially the same amount of information as the 60‐channel montage. Their mathematical demonstrations indicate that whole‐head MEG data contain considerably more neural information than whole‐head EEG data and thus likely have higher utility (e.g., better source localization, better functional connectivity measures). Although sparse channel arrays sometimes suffice to test research hypotheses and are often of clinical interest (e.g., assessing gross brain injury on the sports field (Hanley et al. 2017, 2018), for use as a predictive marker in individuals at clinical high risk for psychosis (Mathalon et al. 2025; Hou et al. 2024)), the spatial sparseness of EEG data limits our ability to characterize what we know are very often regionally specific, complex neural circuit processes.

Of note is the growing interest in high‐frequency activity associated with attention and memory (Jensen et al. 2007), object perception (Tallon‐Baudry 1999), maturation of facial expression processing (Bai et al. 2025), and associated with local and long‐range communication (Canolty et al. 2012, 2006; Osipova et al. 2008). More work is needed to characterize the maturation of high‐frequency neural activity in order to better understand typical and atypical high‐frequency activity in adults (e.g., Edgar et al. 2016; Berman et al. 2015). Examining visual cortex gamma‐band activity in response to visual stimuli and tasks known to produce visual cortex gamma‐band activity (Gray and Singer 1989; Muthukumaraswamy et al. 2010; Gaetz et al. 2012; Muthukumaraswamy and Singh 2013) observed the expected gamma‐band condition effects much more often for MEG than for EEG and reported occipital MEG sensor SNR significantly higher than that for EEG occipital electrodes. Muthukumaraswamy and Singh hypothesized that this difference is due to less contamination of muscle artifact for MEG than EEG, with MEG sensors less prone to long‐distance field conduction effects. For example, EEG studies have shown that even central EEG electrodes are contaminated by large distal muscle activity (Goncharova et al. 2003; Whitham et al. 2008, 2007), with a reduction in neural activity above 20 Hz in EEG sensors when EEG is recorded during complete neuromuscular blockade (Whitham et al. 2008, 2007). With respect to infant recordings, our experience has been that the muscle activity associated with an infant sucking a pacifier (used to calm an infant during a task) has little effect on MEG sensors while introducing large amplitude electromyogenic noise in EEG sensors.^2^

Many clinical uses of EEG are well established, including diagnosis of epilepsy and sleep disturbances, with new applications emerging such as ambulatory EEG and EEG in a home setting (Van Stigt et al. 2023; Laguna et al. 2023; Biondi et al. 2022; Lau‐Zhu et al. 2019). For clinical applications, a case for a very targeted data collection and analysis strategy (e.g., focusing on a few EEG or MEG channels) can sometimes be made (Edgar et al. 2018; Huang et al. 2003). The recent renaissance in EEG is notable, in particular the development of mobile EEG systems (able to tolerate motion Bleichner and Debener 2017), portable EEG systems (small physical size, including EEG devices with wireless capability (Debener et al. 2012)), and transparent EEG systems (portable, motion tolerant, self‐applicable, easy to wear, and unobtrusive (Bleichner and Debener 2017)). Although enabling more studies outside the laboratory, these applications are usually low‐density (<< 64 channels; e.g., see (Niso et al. 2023; Jacobsen et al. 2025)), thus providing even lower‐dimensional data, severely limiting suitability for source localization. As an example of cutting‐edge work in this area is the use of high‐density EEG infant caps in preterm infants in the Neonatal Intensive Care Unit (Kjeldsen et al. 2025; Maitre et al. 2020). A major limitation of MEG (using either SQUID and OPM technologies) is that recordings in the field are not possible.

Of note is the need to distinguish between the richness of MEG versus EEG data in general from the accuracy of MEG and EEG data as a basis for localizing brain activity in specific regions. Studies have shown that MEG and EEG can have comparable accuracy in localizing a single focal source (e.g., early event‐related responses in some primary sensory areas (Cohen et al. 1990; Leahy et al. 1998; Sharon et al. 2007; Komssi et al. 2004)). For bilateral auditory cortex activity, however, the biophysics and the evidence favor MEG over EEG. MEG is preferentially sensitive to superficial, tangentially oriented neural currents such as the superior temporal gyrus (STG) auditory generators (Edgar et al. 2003). This is true even in infants and children (Bosseler et al. 2021; Paetau et al. 1995), with the differentiation of left and right auditory cortex important given hemisphere differences in the maturation of auditory encoding processes (Edgar 2021). In contrast, with EEG, the orientation of those bilateral auditory sources typically leads to a single maximum, located somewhat remotely at Cz, depending on the reference, thus making it challenging to differentiate left‐ and right‐hemisphere activity.

EEG papers often infer regional differences in brain activity based on analysis of groupings of spatially adjacent EEG sensor groups. Such an analysis strategy encourages what is often an unwarranted correspondence between a sensor group (e.g., electrodes over frontal cortex) and the brain area near those sensors (frontal lobes). Authors should justify such assumed sensor‐to‐brain correspondences, demonstrating that the sensors provide valid and reliable measures of neural activity from the inferred brain region(s) despite, in general, being sensitive to large portions of the brain. Authors should also demonstrate that their sensor groupings are unbiased, demonstrating (a) across participants and ages that the same neural generator(s) contribute equally to a given sensor group and (b) across participants and ages as well as within an individual that the homologous left‐ and right‐hemisphere generators equally contribute to the respective sensor groups. Such a sensor group approach will often be difficult to defend, given that studies examining differences in the orientation of neural generator activity (often due to individual differences in gyrus anatomy) show large between‐hemisphere and between‐subject variation in the orientation of the neurons giving rise to the EEG and MEG sensor measures (Edgar et al. 2003, 2017). Analyzing data in source space at the individual level and performing group analysis on source‐level data mitigate this difficulty.

The different spatial sensitivity properties of MEG and EEG are notable. For example, whereas in principle MEG is less sensitive to source currents that are strictly radially oriented, EEG can detect neural generator sources of all orientations (Hillebrand and Barnes 2002). One consequence of this is that MEG tends to favor focal and EEG extended cortical sources (Ebersole and Ebersole 2010; Freeman 2009). Furthermore, although both MEG and EEG are most sensitive to superficial sources, in principle EEG is better suited than MEG to detect signals from deep structures (e.g., thalamus or brainstem). These often‐noted differences between technologies, however, are simplistic. First, because of the reduced sensitivity of MEG to radially oriented sources, it is frequently assumed that MEG is insensitive not only to deep sources but to superficial gyral sources. Several studies have investigated this claim. Hillebrand and Barnes (2002) found that source depth, and not orientation, was the main factor affecting the sensitivity of MEG to activity in human cortex. They also noted that, “there are thin strips (~2 mm wide) of poor resolvability at the crests of gyri; however, these stripes account for only a relatively small proportion of the cortical area and are abutted by elements with a nominal tangential component that are highly resolvable due to the proximity to the sensor array.” Second, numerous studies have provided compelling examples of successful MEG differentiation of deep sources (e.g., hippocampus: (Hanlon et al. 2005, 2011; Meehan et al. 2021; Rempe et al. 2025), cerebellum (Samuelsson et al. 2020; Styliadis et al. 2021), and amygdala (Safar et al. 2021, 2018; Leung et al. 2018)). Box 1 further discusses the localization accuracy of EEG and MEG.

BOX 1 **Localization Accuracy of EEG and MEG.**

In many contexts, the desired location accuracy may be beyond what EEG or MEG can achieve, but that is often not the case (for additional discussions (see Marder and Miller 2024; Miller et al. 2007). Skull phantom work using 32 known dipoles found an average spatial localization error of just 3 mm with 122‐channel, 61‐site MEG and 7–8 mm with 64‐channel EEG, the former largely due to a 2.26‐mm error in sensor location quantification (Leahy et al. 1998). These studies used SQUID MEG, and OPM MEG may provide even better accuracy. For many goals, relative accuracy suffices. As an example, the relative locations of the representations of the fingers of one hand in primary motor cortex can easily be distinguished with a small number of scalp EEG electrodes, most placed over the contralateral motor strip, a case in which the experimenter would know in advance where to place the sensors. Second, the approximate locations of distant sources may suffice, such as assessing nodes in a frontal–parietal network. Third, some sources are conceived as patches or sheets of cortex, rather than dipoles, in which case millimeter accuracy may not be important. Fourth, distinguishing time courses of sources or their sensitivity to different experimental conditions may be more important than determining their precise spatial location.

When considering source localization, of note are the differential effects of the tissue boundaries in EEG and MEG. In the spherical head approximation, the conductivity values of the skull and brain and other tissues do not affect the MEG signal at all, whereas they have a substantial effect on EEG (see Sarvas's formula in his 1987 paper (Sarvas 1987)). Even in more realistic head models, using the boundary element or finite element models, MEG is much less sensitive than EEG to the tissue properties (Vorwerk et al. 2025; Haueisen et al. 1997). This is because the skull's conductivity is much lower than the conductivity of the brain tissue, and thus by far most of the volume current is confined within the inner skull surface. As such, for MEG source modeling, one can ignore the complicated substructure and local variations of the skull and treat it as an insulator, this having minimal effect on the accuracy of the MEG forward model (Hamalainen and Sarvas 1987, 1989). In contrast, even in perfectly concentric spherical head models with multiple layers, the EEG potentials on the scalp are highly sensitive to brain, CSF, skull, and scalp conductivity profiles. Scalp EEG depends on the weak current that “leaks through” the skull and reaches the EEG electrodes. Therefore, for EEG source localization it is important to accurately model the substructures and local variations of the skull conductivity distribution, although this is extremely difficult (Leahy et al. 1998), especially for children where tissue conductivity estimates change across development. In addition to the uncertainties related to head modeling, a major challenge for source localization of both MEG and EEG data is the problem of identifying (“de‐mixing”) contributions from multiple simultaneous sources. Due to the spatial spread (“volume conduction” in EEG) of signals originating from even a focal source, the sensor data typically are a weighted sum of contributions from multiple source regions, with the weights unknown. Determining the local extent of a source is challenging for either MEG or EEG, but for many types of studies (including functional and effective connectivity studies) the interest may be in dissociating between contributions from spatially separated sources. For de‐mixing simultaneously active source regions, MEG's insensitivity to radial sources can be beneficial. In particular, whereas in MEG each local region generates a “dipolar” field pattern mainly corresponding to tangential source currents, in EEG the topographic maps strongly depend on the relative size of the radial and tangential components of each regional source (Hämäläinen et al. 1993).

Other ways to evaluate the quality of MEG and EEG source localization have been considered. For example, convergence with brain regions found with hemodynamic (fMRI, PET) or invasive (intracranial) EEG can suggest that assumptions made and results obtained in MEG and EEG source localization procedures are reasonable. However, due to the very different time scales and other physiological characteristics of the signals, there is no guarantee that the same spatial patterns will be found with the different methods (see, e.g., Hillebrand and Barnes 2002). As such, findings from other modalities such as fMRI and PET do not necessarily serve as a gold standard for MEG and EEG localization (e.g., see Sadaghiani et al. 2022).

The common belief that the inverse problem precludes meaningful electromagnetic source localization is unjustified. Ultrasound imaging faces the inverse problem (Goudarzi et al. 2022) without dismissal of the medical images it produces. The number of EEG or MEG sensors and their topographic sampling density will often be less important than the overall spatial coverage (especially for deeper sources), with sensor placement evaluated based on the anticipated source strength, location (including depth), orientation, and areal extent of the neural generator(s). Source‐analysis algorithms differ considerably, with differing sensitivities to the number and locations of sensors. Source models may involve one‐axis dipoles, three‐axis dipoles (what BESA software calls regional sources), or cortical sheets. Source analysis may rely on sets of pre‐specified targets, selected on the basis of a standard atlas or subject‐specific sMRI or fMRI information, rather than asking the algorithm to model location in addition to orientation and/or time course. Methods exist to produce distributions of candidate source locations (e.g., Huang et al. 1998, 2005). For participants of any age, no single minimum number of sensors can be specified as a prerequisite to EEG or MEG source analysis. The chosen number and placement of the sensors in a given study will affect the spatial capabilities of source localization, but the temporal resolution advantages of EEG and MEG do not depend on those.

For source reconstruction, although co‐registration to each child's individual sMRI is preferred (and needed for multimodal studies, for example relating neural activity to gray‐matter cortical thickness), some studies have found that the use of age‐matched MRI templates is often successful (Douw et al. 2018; Holliday et al. 2003). For infants or individuals with high support needs (e.g., individuals with autism who have a low IQ and are nonverbal), it can be difficult to obtain structural MRI data. As a result, researchers frequently rely on standardized brain templates or surrogate MRIs. Unfortunately, most widely used neuroimaging templates, such as MNI or FreeSurfer's fsaverage, are based on adult anatomy and are therefore not well‐suited for modeling the infant brain. Infant heads and brains differ from those of adults in terms of overall size, cortical folding, skull thickness, fontanels, and cortical thickness, as well as in relative volumes of gray matter, white matter, and cerebrospinal fluid. As discussed in Section 2.2, these anatomical differences lead to errors in the forward model and subsequent source localization, with EEG much more vulnerable than MEG. Although some age‐specific infant templates are available, such as the UNC or dHCP atlases, they have yet to be fully integrated into the leading open‐source MEG analysis packages, and they do not cover the complete range of head sizes in infancy. Moreover, even with age‐matched templates (Power et al. 2025), small errors in co‐registration can produce significant localization errors due to the small size of the infant head. Thus, at present, co‐registration to pediatric template MRIs can be problematic. Rhodes, Rier, Boto, et al. (2025) demonstrated a novel method of co‐registering on‐head OPM data to “pseudo‐MRI” images. These images were created by warping 3D structured‐lights scans (acquired from individual participants) to age‐matched template MRIs. Co‐registration to the pseudo‐MRI images provided source localization accuracy comparable to that of individual MRI scans. This method has since been used for OPM source localization in infants and young children (Rhodes, Rier, Singh, et al. 2025; Safar et al. 2025). This strategy, however, does not provide the ability to assess associations between brain structure and function.

Collecting simultaneous MEG + EEG data is often useful, due to the different spatial sensitivity properties of MEG and EEG, thus providing complementary information (Ebersole and Ebersole 2010; Cohen and Cuffin 1983; Goldenholz et al. 2009; Ahlfors et al. 2010; Piastra et al. 2021). Also, as the degree of interference due to external disturbances and physiological signals of nonbrain origin (eyes, heart, movement) differs for MEG and EEG, combined MEG + EEG recordings can help to dissociate signal artifacts from true brain activity. Because of these complementary properties, simultaneous recording of MEG and EEG can be highly beneficial (Ahlfors 2019). For example, interictal epileptogenic discharges can be identified in some patients with MEG only or with EEG only (e.g., Knake et al. 2006; Ramantani et al. 2006). Maximizing the information content using simultaneous MEG and EEG is important in clinical studies, such as presurgical mapping of epileptogenic as well as intact brain regions (Hari et al. 2018; Stefan and Trinka 2017). Combining MEG and EEG can also improve source localization (e.g., Aydin et al. 2015; Fuchs et al. 1998) and may help distinguish sources (Huang et al. 2003; Sharon et al. 2007). Simultaneous MEG + EEG is expected to be particularly beneficial in exploratory studies in which new findings and phenomena are described (da Silva et al. 2014).

A downside of simultaneous EEG and MEG is the increased subject preparation time required for attaching EEG electrodes. Geodesic EEG nets with saline sponge electrodes (Tucker 1993), which can be applied very quickly, are not well suited for simultaneous use with MEG, because they typically take too much space around the scalp to fit inside the MEG helmet, although this is more feasible in pediatric populations run in MEG helmets sized for adult heads. Long preparation times can affect the vigilance and general state of the child and thus the quality of the neural data and task performance. In infants, given a very short period when the infant is awake and oriented, obtaining simultaneous EEG and MEG is very difficult. A minor disadvantage of simultaneous EEG and MEG recording is that even thin EEG electrodes under a fixed MEG helmet slightly increase the distance between the child's head and the MEG sensors, thereby reducing the strength of the MEG signals. The choice of whether to undertake simultaneous EEG with MEG in clinical or research studies depends on the study goals.

### 2.3 Resting‐State Neural Activity: An Example Demonstrating the Need for High‐Dimensionality Neural Measures With Good Spatial Resolution When Studying Brain Maturation From Infancy to Late Adolescence

This section focuses on resting‐state (RS) measures to demonstrate the need for brain imaging methods with high information content in order to understand how the human brain is constructed and how it matures.

**FIGURE 2:**
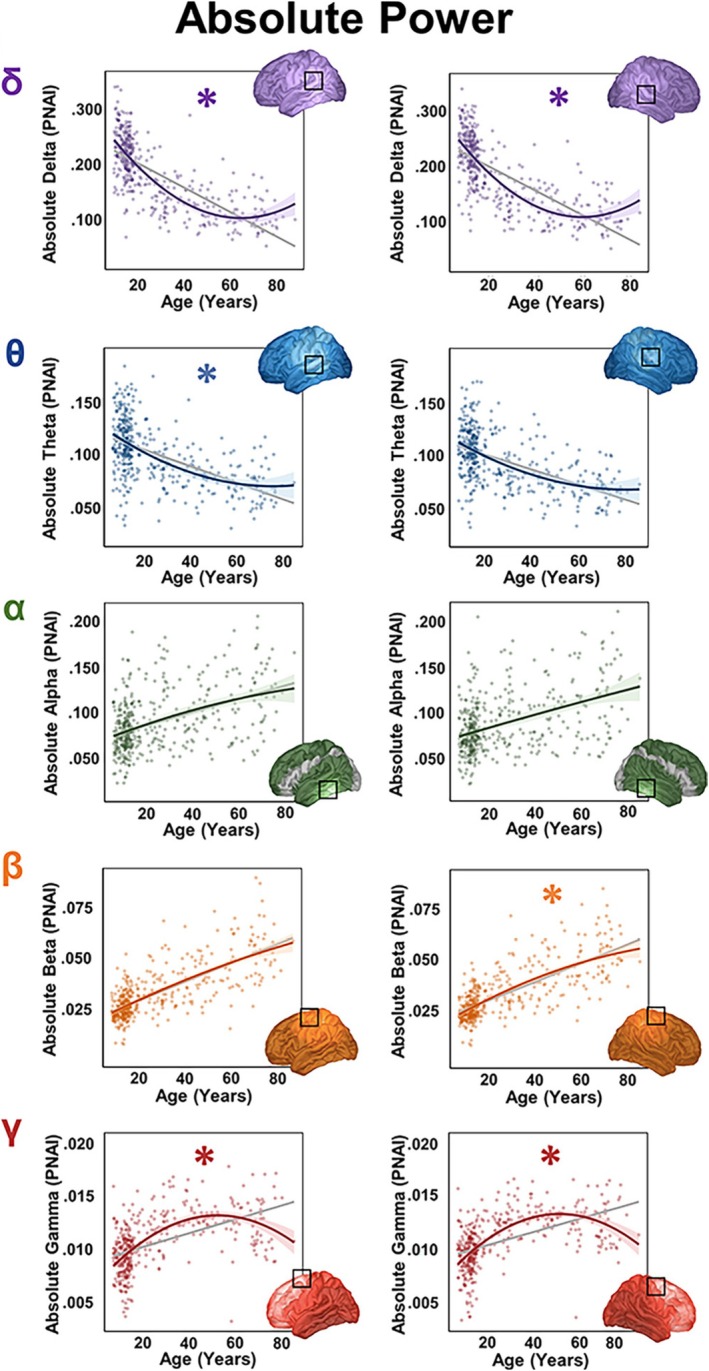
Scatterplots displaying the peak values of the significant main effect of age and power with linear (gray) and quadratic (color) fits for each significant age effect. Asterisks (*) indicate that the quadratic fit is significantly better than the linear. The box on each brain represents the peak of each cluster from which data were extracted. The dots on the scatterplots represent individual participants, with the colored line showing the quadratic fit and the shaded portion representing SE. The color distributions correspond to the canonical frequency band shown (delta‐purple, theta‐blue, alpha‐green, beta‐orange, and gamma‐red). Absolute power in pseudo neural activity index (PNAI) units is plotted on the *y*‐axis, and age is plotted on the *x*‐axis. Left‐hemisphere clusters are in the left column and right‐hemisphere clusters are in the right column. Image from Rempe et al. (2023) and used with permission.

There is a very large EEG and MEG literature on the developmental trajectory of RS neural activity. Studies dating to the 1940s demonstrate changes in RS EEG from birth through adulthood, with age‐related decreases in delta and theta activity and age‐related increases in alpha, beta, and gamma activity (Gibbs and Knott 1949; Penuel et al. 1955; Matoušek and Petersén 1973; Eeg‐Olofsson et al. 1971; Gasser et al. 1988; Edgar, Khan, et al. 2015; Fisch and Spehlmann 1999; Schomer and Lopes Da Silva 2017; Klimesch 1999; Cragg et al. 2011; Petersén and Eeg‐Olofsson 1971; Clarke et al. 2001; Katada et al. 1981; Matsuura et al. 1985). Within a frequency band, of note are regional/functional differences. For example, within the alpha band (8–13 Hz in adults and lower in children), there is a need to differentiate between maturation of parietal‐occipital alpha rhythms modulated by closing and opening the eyes (Klimesch 1999; Adrian and Matthews 1934; Berger 1929), sensorimotor alpha rhythms modulated by somatosensory input and movements (mu rhythms) (Salmelin and Hari 1994; Tiihonen et al. 1989), and superior temporal gyrus alpha rhythms modulated by sound (Salmelin and Hari 1994). Unfortunately, across decades, many studies have ignored the complexity of RS neural activity, including spatial variation, temporal variation on the scale of seconds, minutes, or hours, and longitudinal trajectories. MEG studies are beginning to address this complexity head on (Hunt et al. 2019; Rempe et al. 2023). As an example, (Rempe et al. 2023) showed brain‐region and sex differences in maturation of RS neural activity in a MEG source‐localization study assessing RS activity across the lifespan (433 individuals 6–84 years). Figure 2 (from Rempe et al.) shows that whereas the lower delta and theta frequencies showed a negative correlation with age, the higher alpha, beta, and delta frequencies correlated positively with age. These correlations were further probed with hierarchical regressions, which revealed significant nonlinear trajectories in several brain regions. To our knowledge, no developmental or aging EEG studies have provided RS findings with comparable spatial resolution.

Recently, scientists have also called into question longstanding RS EEG and MEG findings, noting that most RS power‐spectrum analyses conflate two RS brain processes: aperiodic background activity (contributing power across all frequencies if decomposed into sinusoids) co‐existing with periodic oscillations (e.g., actual neural oscillations visible as peaks in the power spectrum, such as the RS dominant oscillation) (He 2014; Freeman and Zhai 2009; Donoghue, Haller, et al. 2020). A growing literature demonstrates the need to distinguish these, specifically to parameterize the RS power spectrum in order to accurately characterize RS maturational changes separately for aperiodic and periodic power (Donoghue, Dominguez, and Voytek 2020; Ostlund et al. 2022; He et al. 2019), with power values in brain space preferred to those in sensor space (Donoghue et al. 2022).

For parameterized RS data, of note is the benefit of neurophysiological measures that are reference‐free. Measurement of the exponent (slope) of the resting‐state power spectrum is hypothesized to provide a non‐invasive measure of the neural‐circuit excitatory:inhibitory (E:I) balance (Gao et al. 2017). Valid determination of this value using EEG sensor measures is complicated by the fact that different referencing strategies, such as bipolar vs. common average, produce very different exponent estimates (Shirhatti et al. 2016). The RS power spectrum exponent and offset parameters can be more directly measured via source‐space analyses.

Describing maturational change in neural activity is of growing interest, including parameterization of the RS power spectrum in children. Most RS parameterization efforts have so far parameterized the EEG sensor power spectra, which reflect activity from multiple brain regions for discussions of the problems associated with this approach (see Edgar et al. 2003, 2017; Hoechstetter et al. 2004; Scherg and Berg 1996; Scherg and Picton 1991). The mixing of activity from multiple brain sources due to volume conduction (Nunez and Srinivasan 2006; Srinivasan et al. 2007) has been especially severe due to most studies obtaining power spectra from an average of EEG channels (Karalunas et al. 2022; Tröndle et al. 2022) or via two or more regional clusters of EEG sensors (Tröndle et al. 2022; Cellier et al. 2021; Carter Leno et al. 2021; Rico‐Picó et al. 2023; Favaro et al. 2023; Hill, Clark, et al. 2022; Schaworonkow and Voytek 2021). Understandably, the EEG cluster studies have produced inconsistent results: Rico‐Picó et al. (2023) and Schaworonkow and Voytek (2021) observed statistically significant regional differences in the aperiodic measures, whereas several other studies did not observe significant regional differences (Cellier et al. 2021; Favaro et al. 2023; Hill, Clark, et al. 2022; McSweeney et al. 2023) or did not statistically assess regional differences (Carter Leno et al. 2021).

Recent studies have empirically demonstrated the difficulties interpreting sensor findings because, due to volume conduction, sensor measures reflect activity from multiple brain areas. Schaworonkow and Nikulin (2022) illustrated this with RS activity by showing how, due to volume conduction, RS alpha activity from occipital, sensorimotor, and superior temporal gyrus neural sources is mixed at the level of the EEG/MEG sensor. As examples, they showed that central sensors often include bilateral activity from all those brain regions and that at frontal EEG sensors the contribution of occipital and sensorimotor neural generators can be as high as 75%, making inferences about the activation of frontal cortex based on frontal electrodes problematic and likely inappropriate. They also noted that the contribution of activity at the sensor level from different brain regions likely changes over time (seconds, hours, days, years) and that between‐subject differences in the brain areas contributing to individual sensors or sensor ROIs are to be expected. Shirazi et al. (2025) used ultrahigh‐density electrocorticography (activity from a 3‐by‐3 cm^2^ patch of cortical surface) to compute the activity that would be observed at 207 EEG scalp electrodes. They concluded that, “The results from the simulated EEG showed that the μECoG activity can be detected in very far EEG electrodes as well as the close‐range EEG electrodes. This finding challenges the notion of attributing the EEG channel activity to the closest cortical area, which is unfortunately a common method in EEG research,” and that, “These findings underscore the critical importance of source‐level analysis methods for accurate interpretation of EEG data and suggest that channel‐level approaches may fundamentally misattribute the cortical origins of observed electrical activity.”

**FIGURE 3:**
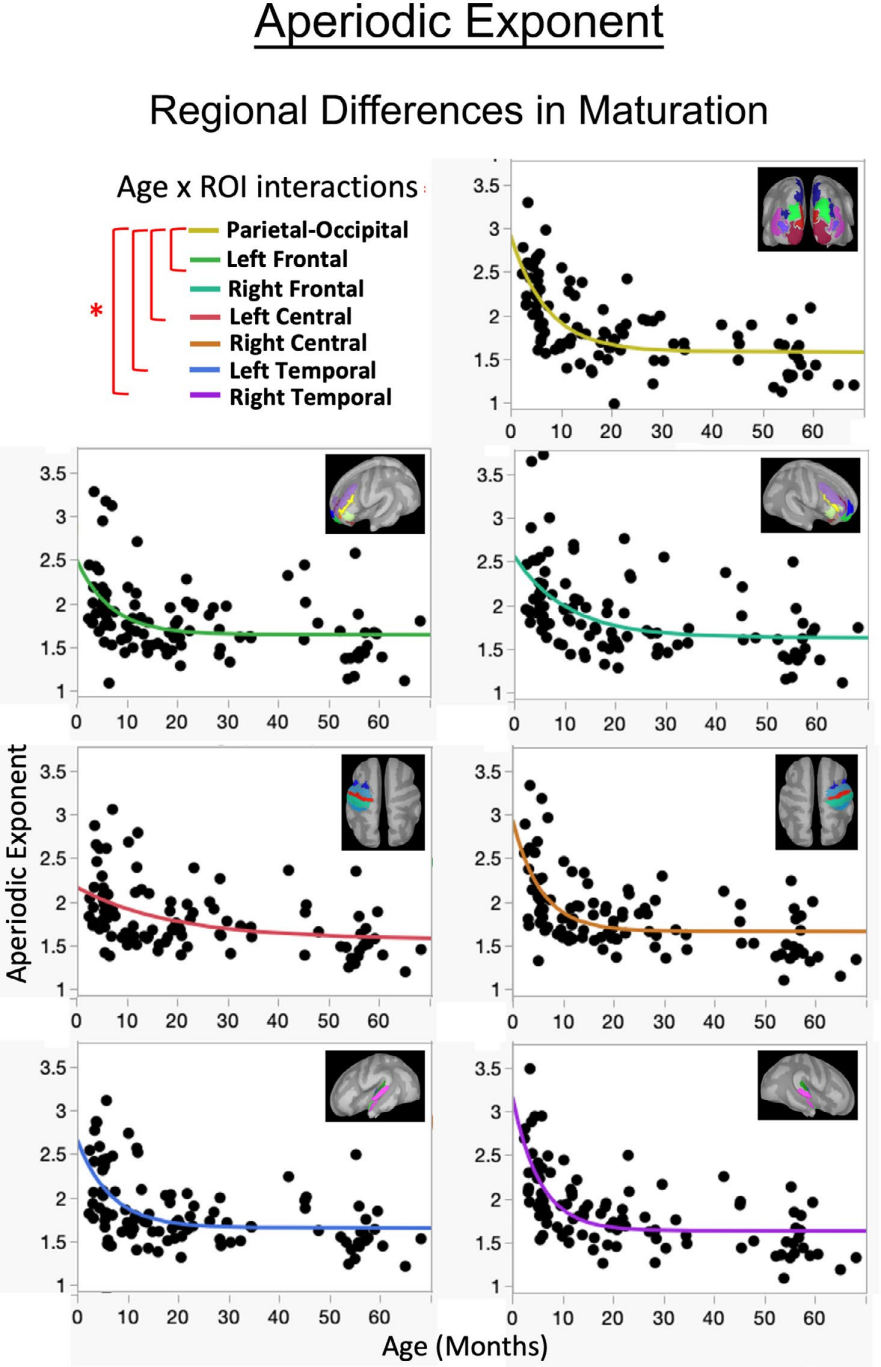
Plots show the relationship between age and the resting‐state aperiodic exponent for the seven examined brain regions. The red lines indicate the statistically significant differences between the parietal‐occipital region and the other brain regions for exponent and age associations. Image from Green et al. (2026). Asterisk and brackets indicate where there were significant differences (*p* < 0.05) between the parietal‐occipital region and the six other brain regions.

For all the reasons discussed in this Section, the difficulties associated with interpreting RS brain activity at the sensor level are many. For the field to advance, scientists and clinicians should make explicit the justification for their methods, ensuring that datasets not only enhance our understanding of brain development in health and disease but also make efficient use of the time‐intensive process of collecting neurophysiology data. EEG and MEG analysis in source space, in contrast to sensor space, are designed to identify specific brain areas and distinguish between them, minimizing blurring. To our knowledge, only three pediatric MEG studies have explored brain area‐specific differences in the aperiodic exponent and offset measures in source space, all reporting spatially dependent differences (Vandewouw et al. 2024; Green et al. 2026; McNamee et al. 2026). For example, Vandewouw et al. (2024) reported regional differences in age and aperiodic exponent and offset associations in 69 children and adults 1–38 years old. As shown in Figure 3, Green et al. (2026) observed in 107 typically developing infants 2–68 months that RS aperiodic measures as well as their maturation differed across the seven brain areas examined.

Adult studies have also reported regional differences in RS neural activity and demonstrate the need for spatially differentiated measures to better understand brain organization. As an example, building on a model of brain organization that postulates a dominant gradient in cortical features across sensorimotor and transmodal areas (Huntenburg et al. 2018), Mahjoory et al. (2020) used RS MEG recordings (*N* = 187 adults) and distributed source localization to show that the dominant periodic peak frequency decreases along a posterior‐to‐anterior axis, following a global hierarchy from early sensory to higher‐order areas. They also showed that the spatial gradient of the peak frequency was anticorrelated with cortical thickness, in effect providing a proxy for cortical hierarchical level. This study demonstrates what is possible with respect to understanding core organizational features of the brain when adequate spatial resolution is available.

With respect to pediatric clinical populations, MEG studies of children with a mild traumatic brain injury (mTBI) are of note. In two recent RS MEG studies (Edgar et al. 2025; Huang et al. 2020), differences in brain activity between children with and without a mTBI were found to be spatially specific. As an example, in a study examining adolescents with mTBI, Edgar et al. (2025) found that group differences in resting‐state beta‐band activity were specific to the superior frontal gyrus, right temporal pole, and right central sulcus.

In sum, given regional differences in the maturation of infant brain structure and chemistry (Bethlehem et al. 2022; Gilmore et al. 2007; Gogtay et al. 2004; Deoni et al. 2015, 2011; Ouyang et al. 2019; Tyzio et al. 2006; Porges et al. 2021; Basu et al. 2021; Ben‐Ari 2014; Yanez Lopez et al. 2021), area‐specific differences in RS activity are almost certain, with age‐varying associations between RS activity and brain structure and behavior very likely. High‐dimensional, whole‐head measures of neural activity that support source‐localization analyses are best suited for such research.

### 2.4 Pediatric MEG: Four Factors That Limit Progress

Although the advantages of SQUID MEG in studying the developing brain are clear, four key factors limit progress: the cost of MEG, the perceived difficulty of multisite MEG studies, the need for more MEG sites, and the need for more scientists trained in source localization methods.

Regarding the cost, many grant reviewers tend to perceive MEG studies as too expensive relative to EEG studies. This premise merits discussion, as it implies a false equivalence between EEG and MEG, and it does not consider the costs of MEG studies relative to the costs of other functional imaging studies, most prominently fMRI.

The financial cost of operating an EEG or MEG lab is substantial, with the costs of setting up a high‐end EEG lab over $300 K USD for a 128‐channel system with multiple EEG caps, an electrode‐location digitization system, data acquisition and data analysis computers and software, and a shielded room (somewhat less without a shielded room). The initial cost of setting up an MEG lab can be 10× that of an EEG lab, consisting of an MEG system, a magnetically shielded room, a digitization system, data collection and analysis computers and software (although most MEG systems also include full EEG systems, valuable for clinical epilepsy cases). The higher financial costs associated with establishing a SQUID MEG laboratory do not end after the lab is set up, with many laboratories spending money on liquid helium (to cool the SQUIDs), perhaps hiring a full‐time technologist, and paying for a yearly service contract. However, the helium operating cost has become less of a problem, as most new SQUID MEG systems incorporate an integrated helium recovery system. The lower cost of purchasing and maintaining an OPM system is discussed in Section 2.5.

The differential financial costs of EEG and MEG research examinations are also of note. EEG research exams are generally almost free, given minimal costs for EEG supplies (e.g., electrode paste or potassium chloride solution). In contrast, most MEG centers expect researchers to pay a substantial machine‐time charge, sometimes as high as the cost of an MRI exam (e.g., between $300 to $700 USD an hour), to recover the cost of purchase and support of the system. In addition, if source localization of EEG or MEG is desired, this ideally includes structural MRI, adding to the cost per participant. In current practice, EEG is a much cheaper technology than MEG.

Importantly but commonly ignored, the financial cost should be considered against the amount of information generated. The advantages of MEG over EEG were reviewed above. Although a low‐density EEG study may be nearly cost‐free to the investigator, the amount of neurophysiological information gained will be far less than that of MEG, especially if structural MRI is also obtained to accompany the MEG data. Furthermore, the high dimensionality of MEG data, combined with its superior spatial resolution, will likely be more valuable than EEG data for future analysis methods yet to be developed. This long‐term advantage is particularly interesting given the growing use of already published datasets for new studies. In sum, although the financial costs of MEG are currently well above those of EEG, they are comparable to those of fMRI research, and the clinical and research yield from MEG will often justify its higher costs, given what it offers in temporal and spatial precision.

A second factor limiting progress is the perception that multisite MEG studies are difficult, based on assumptions that are no longer true or are readily addressable. Multisite studies are needed to establish large sample datasets as well as to achieve adequate representation of diverse rearing environments and of racial, ethnic, and socioeconomic variance. One barrier to such datasets is that there are relatively few pediatric MEG sites that provide recording equipment for smaller heads, as well as variability in MEG systems across sites. That technology is rapidly developing. Another barrier to multisite studies has been difficulty in processing data in a consistent way given that MEG datasets were stored using proprietary data formats requiring custom preprocessing prior to direct comparison. Fortunately, as part of the open data movement, all MEG vendors agreed to make their proprietary data formats readable for developing open‐source software packages. Accordingly, the availability and growing use of open‐source software that supports analysis of MEG data from different systems has removed this barrier. Importantly, differences in sensor type and sensor configurations across vendors are accounted for in the source‐analysis algorithms, thus eliminating a prior barrier to multisite studies. The most widely used open‐source packages include Brainstorm, MNE, and FieldTrip. Commercially available software also can read raw MEG data files from multiple vendors (e.g., Curry, BESA). These developments put MEG on the same plane as MRI, where there are a very small number of dominant hardware vendors, each with their own approach to gradient and head coils as well as proprietary sequences, yet data can readily be harmonized. Numerous techniques have been developed to harmonize multisite data, many of which are based on the ComBat algorithm (Maikusa et al. 2021; Beer et al. 2020). Although there are no published reports applying harmonization algorithms to MEG data, there is ongoing work of the ENIGMA MEG working group, part of the larger ENIGMA consortium (Nugent, in preparation) that combines multisite, multivendor data and harmonization to achieve a large sample dataset. Large‐sample (mostly adult) MEG resting‐state and task datasets are also available from CamCAN (https://opendata.mrc‐cbu.cam.ac.uk/projects/camcan/), OMEGA (https://www.mcgill.ca/bic/neuroinformatics/omega), and POND (https://pond‐network.ca/).

Another issue for multisite studies is that the nature of raw MEG data differs for different types of sensors, for example planar vs. axial gradiometers. However, the properties of these different sensor types are well understood and accounted for in the analysis packages. Accordingly, near‐identical source‐analysis results have been obtained in a human phantom study of three commercially available MEG systems representing most of the vendor market (Weisend et al. 2007).

A general challenge for multisite MEG studies is recording‐system quality in the form of external noise level, stability of sensors, external noise sources (local noise environment, mitigated by the quality of the shielded room), and data acquisition procedures. Depending on the facility setting (in a busy hospital unit vs. a dedicated wing of a building low in acoustic and electromagnetic noise by design), sites may differ in environmental noise. This concern is addressable via a variety of signal processing tools now readily available in both open‐source and commercial MEG analysis packages. Differences in data acquisition procedures across sites can be managed via a standardized protocol, which has become common in multisite studies. In short, the primary challenges for multisite MEG studies are readily addressable (e.g., Vaghari et al. 2022; Hughes et al. 2019).

A third and fourth limitation that constrain the more general use of MEG is the relatively small number of scientists working in this area. Given the rapid growth in clinical MRI sites in the 1990s (in large part due to the growing number of clinical indications for MRI), many scientists were trained in MRI/fMRI methods (e.g., onsite, via summer FSL and SPM workshops, and via university courses). These scientists went on to fill a growing number of positions at medical centers and universities and trained more MRI and fMRI scientists. This growth has benefited from many MRI systems being already in place for clinical sMRI services. In contrast, given only two established clinical indications for MEG (presurgical planning for epilepsy and mapping of eloquent cortex), there are relatively few MEG centers and thus fewer opportunities for students and colleagues to train in MEG. Nevertheless, given that MEG clinical assessments rely on source localization (with the neurosurgeon needing millimeter precision), this clinical need has helped drive the development of source localization methods (benefitting EEG as well as MEG) over the last 30+ years.

Training in MEG occurs primarily via an apprenticeship model, a student training with a senior investigator for years before becoming proficient. Additional clinical indications for MEG will increase demand for MEG centers, with more sites for students to train in, but the addition of new clinical indications is several years in the future. (Clinical applications with individuals with concussion and individuals with autism spectrum disorder are leading contenders.) To make faster progress in understanding brain neural activity in children, we encourage the field to re‐think how electrophysiology studies are conducted, with funds allocated to establish more MEG sites and to develop programs to train more scientists in the use of MEG and EEG source localization methods. There is already evidence that some countries have adopted these two priorities. For example, China recently sited several MEG systems (SQUID and OPM) and provided funds for Chinese scientists to train in MEG and for Chinese engineers to help develop the technology (e.g., Ren et al. 2025; Wang et al. 2024).

### 2.5 Optically Pumped Magnetometers (OPMs): The Next Frontier

There are several limitations to SQUID technology in currently available MEG systems. As discussed above, SQUID MEG laboratories are costly to set up and maintain. Another limitation is that SQUID systems use a fixed‐size helmet holding the sensors, and most current MEG laboratories use systems sized for adults, generally designed to accommodate the 95th percentile male head (Marinkovic et al. 2004). They can accommodate smaller heads, but in children the SQUID MEG sensors can be located a considerable distance from the head surface. Because the strength of MEG signals decreases with the square of the distance from the source, on‐scalp OPM sensors can provide much higher signal magnitudes than SQUID MEG systems designed for adult head sizes. SQUID MEG systems optimized for infants and toddlers have recently become available (for review (see Chen et al. 2019)), such as the Artemis 123 system (Tristan Technologies Inc.) designed to accommodate the median 3‐year‐old head circumference (Edgar, Murray, et al. 2015; Roberts et al. 2014). However, even the systems optimized for young children use a fixed‐size helmet, which is not optimal for head sizes that are growing during development. Moreover, current pediatric helmets remain larger than is optimal for young infants. Thus, there remains a challenge in obtaining the optimal signal from each child across development.

A related limitation of SQUID‐based MEG is that the helmet is necessarily stationary. Thus, participant head movement leads to a reduction in data quality analogous to that affecting MRI measurements, and movement is a particular challenge in awake infants and young children. In modern SQUID‐based MEG systems, this is addressed by continuously recording from head position indicator coils and using tools such as signal‐space separation (SSS) and tSSS (temporal SSS) to adjust signals for head movement following the recording (Taulu and Hari 2009; Taulu et al. 2004; Taulu and Simola 2006; Wehner et al. 2008; Larson and Taulu 2017). Nonetheless, head movement results in changes in the distance between the sensor and source, leading to variability in signal‐to‐noise ratio across the data collection session and constraining the desired behavior of the participant.

Emerging OPM technology provides a promising avenue with which to address these limitations of SQUID MEG. Briefly, OPM sensors are a form of compact quantum sensors that measure the magnetic fields associated with neural activity but do not require liquid helium (Tierney et al. 2019; Boto et al. 2017; Zhao et al. 2023). Each sensor cartridge can be placed directly on the scalp surface, minimizing the distance between source (brain) and sensor. Tremendous progress has been made in fabricating small and lightweight OPM sensors of various types,^3^ which has led to the development of wearable OPM MEG helmets. Whole‐head OPM arrays with more than 200 channels have been demonstrated, and the newest devices are beginning to match the density of commercially available SQUID‐based MEG systems.

The current generation of OPM sensors enables many different implementations, including stationary helmets affixed to a scanner bed (Bardouille et al. 2024), wearable rigid helmets of different sizes (Hill et al. 2020; Rhodes, Rier, Singh, et al. 2025), and EEG‐like caps (Corvilain et al. 2025; Power et al. 2025). Rigid structure helmets may be 3‐D printed based on individual MRIs (Boto et al. 2018), thus enabling whole‐head arrays that correspond closely to individual head shapes. However, this approach is extremely expensive and not feasible for routine studies. Given the inability of infants to fully support their own heads, OPM helmets often require some form of weight support, but this can be as simple as leaning against a caregiver.

**FIGURE 4:**
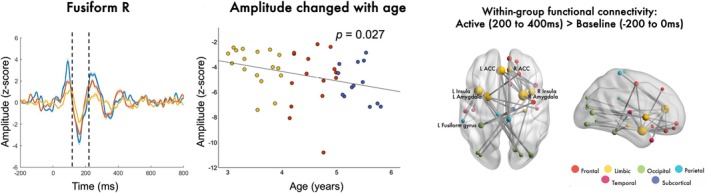
Results from a recent OPM study in which children aged 3–5 saw images of emotional (happy and angry) faces. Left panel: The M170 evoked response to faces in the right fusiform gyrus, in different age groups. Middle panel: M710 amplitude, averaged across left and right fusiform gyri, as a function of age. Right panel: Mean (across all participants) functional connectivity in response to face presentation relative to a preceding baseline period. Image from Safar et al. (2025) and used with permission.

The ability to place OPM sensors directly on the surface of the scalp is a game‐changer for pediatric neuroimaging. Notably, wearable OPM allows participants an impressive range of motion without seriously impacting data quality, provided the head does not move relative to the sensors (O'Neill et al. 2025; Seymour et al. 2021). This is particularly valuable in pediatric neuroimaging, where young children often struggle to remain still throughout a testing session. Indeed, recent work using wearable OPM has demonstrated success in testing children and toddlers (Vandewouw et al. 2024; Rhodes, Rier, Singh, et al. 2025; Rier et al. 2024; Safar et al. 2025) and infants as young as 1 month of age (Corvilain et al. 2025). For example, Figure 4 (adapted from Safar et al. (2025)) shows neural responses to faces in a group of children aged 3–5, including age‐related changes in region‐specific evoked responses (left and middle panels) and whole‐brain functional connectivity (right panel). This study demonstrates the suitability of OPM for capturing complex neuronal responses to stimuli in pediatric populations.

The benefits of on‐scalp OPM sensors also bring challenges. Knowing the precise location and orientation of each MEG sensor is required for accurate source reconstruction. The advantage of a fixed array (as in SQUID MEG) is that the sensor positions and orientations can be determined once and remain valid throughout the life of the system. The adaptability of OPM arrays to differing head shapes poses a technical consideration—if the sensor locations and/or orientations are allowed to differs across participants, their positions must be determined for each session. One way this problem can be mitigated is by using rigid helmets of varying (e.g., age‐appropriate) sizes that fix the OPM sensor positions and orientations (e.g., Hill et al. 2020; Rhodes, Rier, Singh, et al. 2025). There are also helmets with adjustable sensors that can be pushed into the helmet until they meet the subject's scalp (e.g., Bardouille et al. 2024). These helmets may incorporate measurement coils that allow the position of each sensor to be detected, or external coil arrays may be incorporated into the helmet for calibration. Finally, there are mathematical algorithms that can be used to determine the positions and orientations of OPM sensors with the help of recorded calibration data. For example, Iivanainen et al. (2022) demonstrated a method that utilizes external field compensation coils and fluxgate measurements to fit the relevant coefficients of a vector spherical harmonic expansion in the area of the OPM array. These coefficients then served as a signal model against which the sensor parameters were fitted. This additional time for calibration is a consideration for pediatric studies.

**FIGURE 5:**
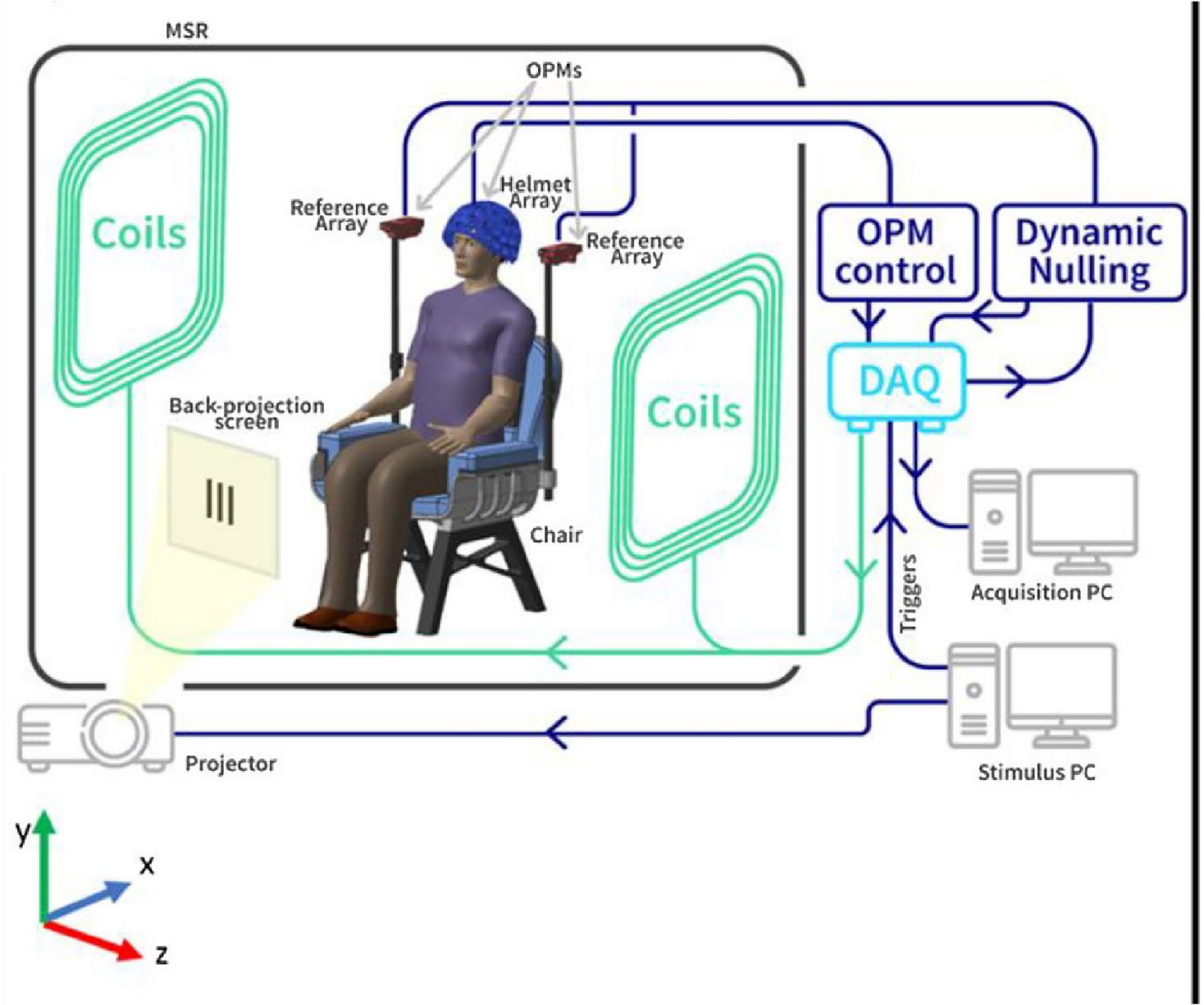
Schematic illustrates OPM recording within a low magnetic field environment between biplanar nulling coils; these in turn are situated inside a magnetically shielded room. DAQ: Data acquisition system. Figure adapted from Hill, Devasagayam, et al. (2022) and used with permission.

Another challenge is the fact that, in addition to sensor location geometry, in some types of OPMs sensor‐specific scalar calibration factors, or gains, may change during a recording session due to the phenomenon of cross‐axis projection error (Borna et al. 2022). To mitigate this issue, careful suppression of the background interference field is necessary. The most common approach to address this problem is by adding active noise cancellation coils to the magnetically shielded room (Holmes et al. 2018; Rea et al. 2021). One solution is to maintain a ~1 m^3^ cube around the participant in which the ambient magnetic field has been minimized using nulling coils, although this limits participant movement to the 1 m^3^ cube. Figure 5 shows a schematic of such a setup as detailed in Hill, Devasagayam, et al. (2022). More recent approaches have incorporated nulling coils on the internal walls of the MSR itself, enabling a larger spatial extent of magnetic field control (e.g., Holmes et al. 2022). Such developments, alongside novel mobile recording platforms (Schofield et al. 2024) have shown promise in allowing movement throughout the magnetically shielded room (Holmes et al. 2023b), though more work in this realm is needed before establishing OPM as a truly ambulatory technology (see also methods in (Tierney et al. 2019; Brookes et al. 2022)). Finally, improvements in the technical performance of the OPM sensors themselves (e.g., noise floor, dynamic range), which do not yet match that of SQUID MEG sensors, will be necessary if OPM technology is to be as useful as current SQUID MEG.

Despite its higher noise floor compared to SQUIDs, OPM MEG holds the promise of increased signal‐to‐noise ratio (SNR) and spatial precision due to closer proximity to the brain, especially in infants and children with smaller head sizes (Brookes et al. 2022). Compared with conventional SQUID MEG, on‐head OPM can deliver equivalent or superior sensitivity to region‐specific evoked and oscillatory responses (e.g., Safar et al. 2024). Moreover, owing to the potentially flexible shape of OPM arrays, judicious placement of sensors to target a particular area can further improve source‐localized SNR (Hill et al. 2024). Rapid technical advances in OPM sensor systems, noise rejection algorithms, and source models are ongoing. Given the tradeoffs between OPM (i.e., higher noise floor but sensors on the scalp) and SQUID MEG (i.e., more sensitive to tiny magnetic field changes but sensors are more distant from the scalp), direct comparisons are needed.

The cost of an OPM system largely depends on the price of a magnetically shielded room and the number of OPM sensors. Like SQUID MEG, OPM recordings will require a magnetically shielded room (several hundred thousand dollars), although smaller rooms and even person‐sized enclosures are now available at a reduced cost. Whereas at present the cost of an OPM sensor (providing one to three orthogonal channels at one scalp location) is in the range of $7K to $10K USD, with yet unknown lifespan, it is anticipated that sensor cost will decrease dramatically as volume increases and the technology matures. Because OPM systems are modular, more sensors can be added later, the barrier of entry to using OPM MEG has dropped dramatically. Scientists considering purchasing an OPM system at the present time still need to consider dedicated staff, with the availability of a commercially available plug‐and‐play OPM system requiring little ongoing onsite support several years in the future. The full cost of an OPM system for a usable lifetime equivalent to that of a SQUID MEG system (decades in most cases) remains to be determined. However, as OPM technology is inherently different than SQUID systems, other factors such as simplified reparability and parts replacements and appropriate service contracts can ensure system uptime of OPM MEG systems.

At present, the OPM arena is diverse and evolving rapidly, with some laboratories focused on collecting dedicated research data and others more active in testing and validating OPM sensors and associated hardware, as well as optimizing OPM data collection and analysis methods. With respect to the clinical use of OPMs (e.g., identifying seizure onset zone(s)), of note is a 2023 commentary from the American Clinical MEG Society (ACMEGS) concluding that, “Based on the engineering obstacles and the clinical tradeoffs to be resolved, the assessment of experts suggests that there will most likely be another decade relying solely on ‘frozen SQUIDs’ in the clinical MEG field” (Bagić et al. 2023). This assessment, however, may be overly pessimistic. OPM studies examining brain activity in patients with epilepsy show promising results in adult (Feys et al. 2025; Ren et al. 2025) and pediatric populations (Feys et al. 2023, 2022). Notably, in 2024, China approved the use of OPM systems for clinical work. Several hospitals in China are using OPM systems to plan surgery for epilepsy and brain tumors. It is hoped that studies from China systematically comparing SQUID and OPM clinical results will be forthcoming.

Despite the above hurdles, many scientists are devoting considerable time to developing OPM technology, on their own or through partnering with industry. The ability to place sensors directly on the scalp of participants may revolutionize pediatric as well as adult MEG. Box 2 considers the use of OPM sensors to examine neural activity in more realistic situations than viewing stimuli on a computer screen, such as obtaining brain measures when two humans are interacting (hyperscanning). The ability to use OPMs to obtain measures of neural activity in parts of the body other than the head is also briefly discussed.

BOX 2 **OPMs In Hyperscanning Studies and for Use Outside the Brain.**

Hyperscanning refers to simultaneous recording of neurophysiological activity from the brains of multiple individuals, whether in the same or different geographic locations. Unlike the traditional approach of recording neural responses throughout highly controlled experiments, hyperscanning can be used to capture brain activity during naturalistic, participant‐led social interactions. For example, some studies have recorded simultaneous EEG from two participants engaged in conversation (Douglas et al. 2023; Fachner et al. 2019), musicians playing music together (Lindenberger et al. 2009; Müller et al. 2013), or high school students engaged in classroom learning activities (Dikker et al. 2017). Such applications—which provide unique insights into the neural mechanisms supporting social cognition—are relevant to pediatric neurophysiology, for example examining social processes in children with autism (for a review, see Zhou and Wong 2024).

**FIGURE 6:**
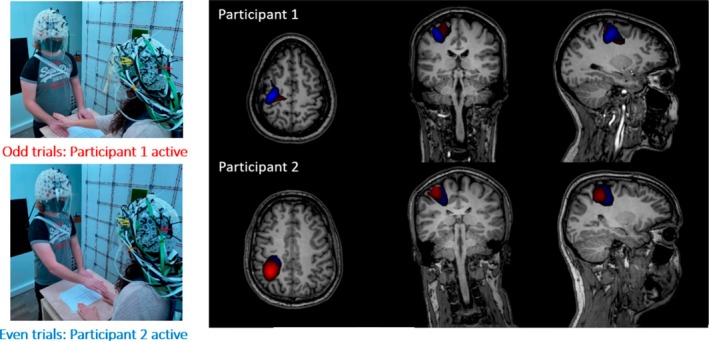
Example of a hyperscanning experiment. Images on the left show two participants engaged in an alternating hand‐touching task while their brain activity is recorded with wearable OPM. The MRI images show source‐localized beta‐band modulation (thresholded to 80% of the maximum value) on odd trials (red: When Participant 1 is active) and even trials (blue: Participant 2 is active). Figure from Holmes et al. (2023a) and used with permission.

OPM MEG is well‐positioned to build on this research. In the first OPM hyperscanning study, Holmes et al. (2023a) recorded from two individuals while they performed interactive hand‐touching and table tennis inside a magnetically shielded room with active field control. As shown in Figure 6, despite task‐induced movements, the authors reported the expected modulation of neural oscillatory activity at the participant level. The insensitivity of OPM‐MEG to movement is a major advantage for hyperscanning over EEG, SQUID MEG, and fMRI, all of which are extremely sensitive to movement. For example, OPM hyperscanning may be more easily performed on young children or patients who find it difficult to remain still throughout an experimental session. More broadly, the reduced constraints on participant movement open the door to novel research areas. For example, it may be possible to perform hyperscanning during nonverbal communication (e.g., sign language), and OPM may enable more naturalistic participant behavior during verbal conversations, in which speech is typically accompanied by physical gestures (Graziano and Gullberg 2018).

Beyond the usual requirements of EEG hyperscanning (see Douglas et al. 2023), the only additional requirement for OPM MEG is a magnetically‐shielded room that is large enough to accommodate the desired number of individuals. By contrast, hyperscanning with SQUID MEG requires either simultaneous recording at two sites (e.g., Ahn et al. 2018; Yoneta et al. 2022) or two systems in the same room (Hirata et al. 2014), which is rarely feasible.

OPMs also present opportunities for measurement of neurophysiology outside the brain. All currently available SQUID MEG systems employ a predefined sensor layout, whereas OPMs may be flexibly arranged to record from other parts of the body. For example, OPMs have been used to record muscle responses in the hand following ulnar nerve stimulation (Nordenström et al. 2024), evoked fields from the retina following light stimulation (Westner et al. 2021), and evoked fields from the spinal cord following median nerve stimulation (Mardell et al. 2024). Remarkably, the latter study source‐localized evoked fields and reported that spinal sources were consistently lateralized to the side of stimulation. This demonstration of accurate spinal source localization hints at the utility of this approach for diverse diagnostic applications.

OPM has even been used to record fetal neurophysiology in utero. By placing OPMs on mothers' abdomens, studies have successfully recorded fetal cardiac rhythms (Batie et al. 2018; Strand et al. 2019; Wurm et al. 2023). Batie et al. (2018) were additionally able to detect cardiac rhythm abnormalities, highlighting the clinical utility of this approach. Future OPM work may substantially expand the clinical and research applications of fetal MEG. For example, SQUID MEG has been used to detect visual and auditory evoked neural responses from fetuses in utero (for a review, see Chen et al. 2019). OPMs offer a lower‐cost method to further explore activity in the developing brain.

It is also worth highlighting that the flexibility of OPM MEG facilitates sensitivity to deep sources in the brain that are less accessible to SQUID MEG, owing to the rapid signal drop‐off between deep brain sources and sensors at the scalp. Tierney et al. (2021) were able to record task‐related activity from the hippocampus using an OPM sensor mounted on a dental mold against the roof of a participant's mouth (although SQUID MEG can successfully record hippocampal activity from the scalp, e.g., Hanlon et al. 2005, 2011; Meehan et al. 2021). This novel application of OPM, alongside the other applications discussed above, demonstrates that OPMs improve study of deep tissue both inside and outside the brain.

## 3 Conclusions

This manuscript focuses on the present and future applications of MEG in pediatric research, emphasizing the practical side of data collection. The advantages and challenges associated with pediatric MEG were discussed from multiple angles, including biophysical, neurophysiological, clinical, and economic aspects. Given the advantages reviewed above of MEG over EEG (simpler source localization mathematical models, higher dimensional data, little effect of open fontanelle and sutures on infant neural measures, and reference‐free neural measures), MEG is often the preferred method for assessing neural activity in children in both clinical and research contexts, including adding substantial value in multimodal imaging. The high dimensionality of MEG data also makes it better suited than EEG to complex spatially resolved analysis methods that are under active development. The promising potential of OPM technology to advance our understanding of neural function in children was also reviewed. Optimizing information acquisition is critical for understanding typical and atypical brain development, and rapid advancements in artificial intelligence are expected to benefit and to benefit from the use of rich MEG datasets and to increase the utility of high temporal and spatial resolution methods such as MEG for improving outcomes across the lifespan.

## Author Contributions

**J. C. Edgar:** conceptualization, writing – original draft, writing – review and editing, project administration, supervision. **T. W. Wilson:** conceptualization, writing – original draft, writing – review and editing, supervision. **S. Taulu:** writing – original draft, writing – review and editing. **A. C. Nugent:** writing – original draft, conceptualization, writing – review and editing. **S. P. Ahlfors:** writing – original draft, writing – review and editing. **T. Kenet:** writing – original draft, conceptualization, writing – review and editing. **Y. Chen:** writing – original draft, writing – review and editing. **A. N. Bosseler:** writing – original draft, writing – review and editing. **H. L. Green:** writing – original draft, writing – review and editing. **E. Heinrichs‐Graham:** writing – original draft, writing – review and editing. **W. Gaetz:** writing – review and editing. **E. Larson:** writing – original draft, writing – review and editing. **F. T. Candelaria‐Cook:** writing – original draft, writing – review and editing. **N. M. Bell:** writing – review and editing. **E. M. Davenport:** writing – review and editing. **B. Howell:** writing – review and editing. **M. Evans:** writing – review and editing. **B. Ahtam:** writing – original draft, writing – review and editing. **M. X. Huang:** writing – original draft, writing – review and editing. **T. W. Ward:** writing – review and editing. **B. Share:** writing – review and editing. **M. Mody:** writing – review and editing, writing – original draft. **J. J. Son:** writing – review and editing. **D. Cellier:** writing – review and editing. **N. A. Peatfield:** writing – review and editing. **T. Bardouille:** writing – review and editing. **L. M. Bailey:** writing – review and editing. **S. Grewal:** writing – review and editing. **K. Pratt:** writing – review and editing. **S. Knappe:** writing – review and editing. **O. Alem:** writing – review and editing. **G. A. Miller:** conceptualization, writing – original draft, writing – review and editing, supervision. **J. Stephen:** conceptualization, writing – original draft, writing – review and editing, supervision.

## Funding

This study was supported in part by National Institutes of Health grant R01MH107506 (J.C.E.), R01AA029605 (J.S.), P50AA022534 (J.S.), NICHD grant R01HD093776 (J.C.E.), NICHD grant R01HD119165 (Y.C.), TALK Supplement Grant GRT‐00003651 (Y.C.), and NICHD grant K08HD114880 (H.L.G.).

## Conflicts of Interest

The authors declare no conflicts of interest.

## Data Availability

Data sharing not applicable to this article as no datasets were generated or analyzed during the current study.

## Endnotes

^1^
For readers with an interest in EEG and MEG instrumentation and technology, Orrison (1995) provided a detailed but accessible review. Schomer and Lopes Da Silva (2017) and Nunez and Srinivasan (2006) provided more extensive reviews of EEG (see also Geselowitz 1970; Geselowitz and Miller 1973; Plonsey 1981; Hämäläinen et al. 1993; Hari and Puce 2023; Rhodes et al. 2024). For reviews of the use of MEG to study clinical disorders (see Rhodes et al. 2024; Dmytriw et al. 2025; Edgar, Guha, et al. 2020). Readers are also referred to papers discussing best practices for data collection, analysis, and reporting for EEG (Picton et al. 2000; Keil et al. 2022, 2014) and MEG (Keil et al. 2022, 2014; Gross et al. 2013; Clarke et al. 2022). Finally, for readers interested in OPM instrumentation and methods, several papers are of note (Tierney et al. 2019; Brookes et al. 2022; Alem et al. 2023); and see references in Section (2.5).

^2^
A noted limitation of the Muthukumaraswamy and Singh study is that the MEG (275‐channel CTF system) and EEG (62‐channel Biosemi Active Two) data were collected on different days and in different laboratories, in part because the investigators did not want the EEG sensors to increase the distance between the participant's head and the MEG sensors. Another limitation was that the MEG and EEG gamma measures were examined at the sensor rather than source level.

^3^
OPM sensors have a chamber containing a vapor of atoms such as rubidium, cesium, or helium. These atoms can be spin‐polarized with light, which makes them sensitive to small magnetic fields. In the presence of a magnetic field, the spin‐polarization changes, and this change can be detected by monitoring the fluctuations of light passing through the atomic vapor. There are a variety of implementations of OPMs (for an overview, see Budker and Romalis 2007), but the type best suited for MEG is the zero‐field magnetometer (Dupont‐Roc et al. 1969). These sensors are among the most sensitive detectors and lend themselves to miniaturization (Schwindt et al. 2004; Shah et al. 2007). Currently, there are several OPM MEG vendors with their unique implementations and this has resulted in different characteristics that impact data quality and system usability. Thus, care should be exercised to not generalize a specific implementation as a feature or limitation of all OPM MEG systems.
